# Supplementary material for: Using Patient-Derived Xenograft (PDX) Models as a ‘Black Box’ to Identify More Applicable Patients for ADP-Ribose Polymerase Inhibitor (PARPi) Treatment in Ovarian Cancer: Searching for Novel Molecular and Clinical Biomarkers and Performing a Prospective Preclinical Trial
Source: Cancers (Basel). 2022 Sep 24;14(19):4649. doi: 10.3390/cancers14194649 (PMC9563731; doi:10.3390/cancers14194649)
Supplement: Supplementary file 1 [file cancers-14-04649-s001.zip › Supplementary Figure Legend.pdf]

Figure Legend

Supplementary Figure S1. The experiment of niraparib and olapalib sensitivity tests in PDX models.

Experimental results of niraparib and olapalib sensitivity tests on PDXs, including response category (the waffle figures, A and C) and best response (%) of tumor volume (the raincloud figures, B and D). Each square in waffle figures represents a PDX model, and the color indicates different response categories: Green-complete remission (CR), blue-partial remission (PR), purple-PR>stable disease (SD), yellow-SD, orange-SD>progression disease (PD), and red-PD, while the data was depicted in the table under the figure. In the raincloud figures, the ‘cloud’ in the picture (upper) is the data core density, shown as the half of the violin diagram, the boxplot (middle) shows quartile and extremum, and the ‘rain’ (lower) is every single value shown as the scatter diagram. The red dots and a line represent the average.

Supplementary Figure S2. Efficacy in PDXs from patients with different mutational characteristics.

The combined tumor volume change (Left volume), the efficacy of each PDX model for one patient (moderate volume), and the change of each corresponding mouse body weight over time (right volume) of all PDX models for one patient in the niraparib and Vehicle groups. Each row represents a patient whose identification is at the left: (A) Patient 20 with a germline BRCA1 mutation and HRD positive; (B) Patient 25 with a somatic BRCA1 mutation and HRD positive; (C) Patient 24 with BRCA wild type and HRD positive; (D) Patient 07 with BRCA wild type and HRD negative. HRD, homologous recombination deficiency. Information for each PDX is on the

right.

Supplementary Figure S3. Genomic analysis of olaparib and niraparib response and non-response groups.

(A,) The copy number variations (CNVs) in the olaparib and niraparib both non-response groups. (B) The number of dysfunction sites in response and non-response groups. (C) The mutation signature analysis in response and non-response groups. (D, E) The onco-Interaction analysis in response (D) and non-response (E) groups.

Supplementary Figure S4. Simulate the NOVA, SOLO I, and PRIMA clinical trials.

(A, B) Simulating NOVA test, in which only patients with platinum-sensitive relapsing were enrolled. PFS (A) and OS (B) were compared between niraparib and vehicle groups. (C, D) Simulating SOLO I test, in which primary patients with BRCA mutation and sensitivity to platinum were enrolled. PFS (C) and OS (D) were compared between olaparib and vehicle groups. (E-J) Simulating PRIMA test, in which only primary patients with stage III/IV and residue tumor after surgery were enrolled. PFS and OS between niraparib and vehicle groups for all patients (E, H), patients with HRD+ (F, I), or patients with HRD- (G, J) were compared.

Supplementary Figure S5. Comparing efficacy between niraparib and TC in PDX

The Best Response (%) and Response Category of control, niraparib, and TC chemotherapy groups in PDXs of naive patients (A, B), HRD+ subgroup(C, D), BRCA mutation subgroup (E, F), or patients simultaneously responded to niraparib, and TC (G, H) were analyzed. TC, Paclitaxel,

and carboplatin chemotherapy; CR, complete remission; PR, partial remission; SD, stable disease; PD, Progression disease.
