# Supplementary material for: Using Patient-Derived Xenograft (PDX) Models as a ‘Black Box’ to Identify More Applicable Patients for ADP-Ribose Polymerase Inhibitor (PARPi) Treatment in Ovarian Cancer: Searching for Novel Molecular and Clinical Biomarkers and Performing a Prospective Preclinical Trial
Source: Cancers (Basel). 2022 Sep 24;14(19):4649. doi: 10.3390/cancers14194649 (PMC9563731; doi:10.3390/cancers14194649)
Supplement: Supplementary file 1 [file cancers-14-04649-s001.zip › Supplementary Figure S4.pdf]

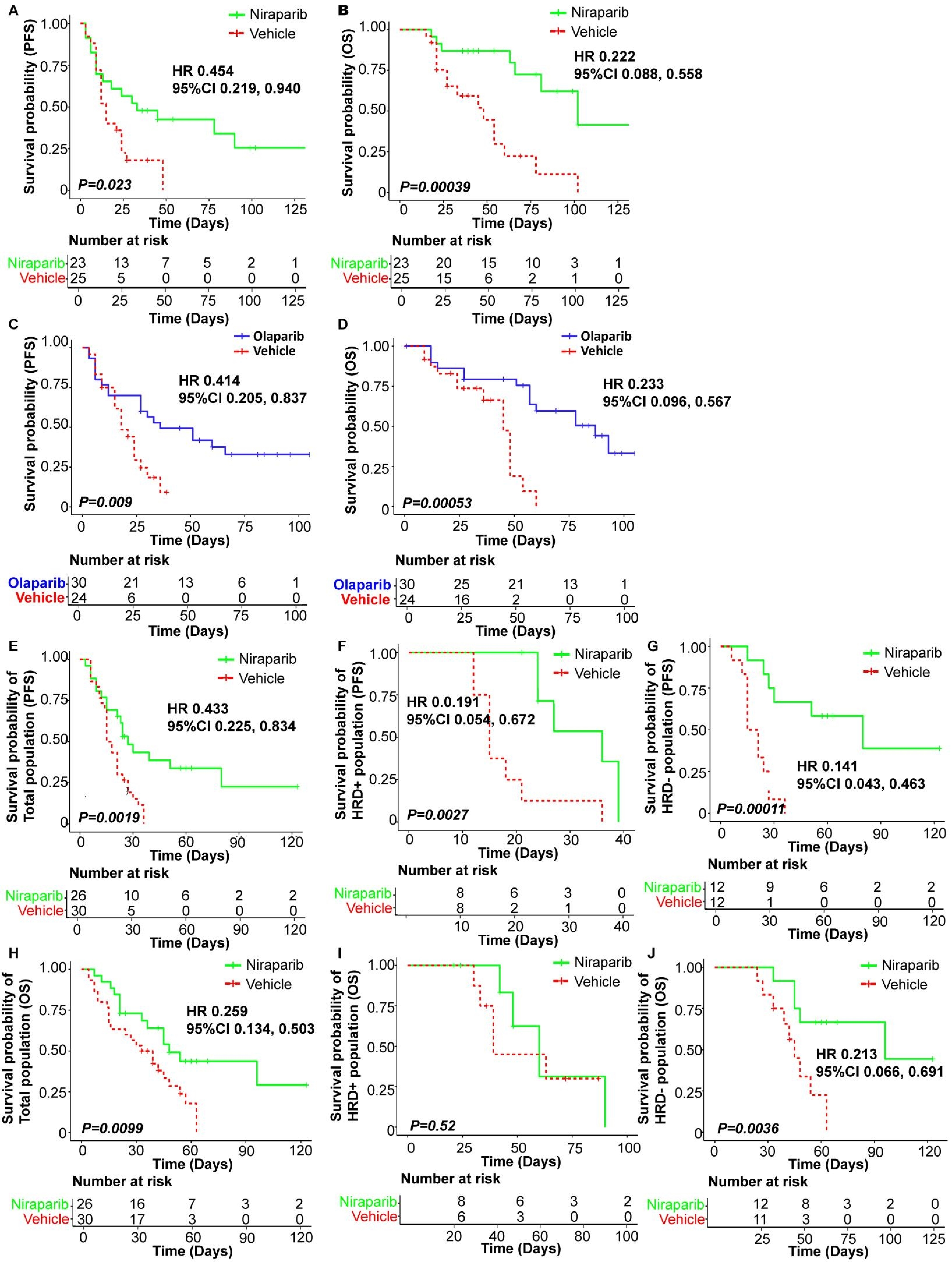

A
Niraparib
Vehicle
HR 0.454
95%CI 0.219, 0.940
P=0.023
Survival probability (PFS)
Time (Days)
Number at risk
Niraparib 23 13 7 5 2 1
Vehicle 25 5 0 0 0 0
B
HR 0.222
95%CI 0.088, 0.558
P=0.00039
Survival probability (OS)
Niraparib 23 20 15 10 3 1
Vehicle 25 15 6 2 1 0
C
Olaparib
Vehicle
HR 0.414
95%CI 0.205, 0.837
P=0.009
Olaparib 30 21 13 6 1
Vehicle 24 6 0 0 0
D
HR 0.233
95%CI 0.096, 0.567
P=0.00053
Olaparib 30 25 21 13 1
Vehicle 24 16 2 0 0
E
Survival probability of Total population (PFS)
HR 0.433
95%CI 0.225, 0.834
P=0.0019
Niraparib 26 10 6 2 2
Vehicle 30 5 0 0 0
F
Survival probability of HRD+ population (PFS)
HR 0.0.191
95%CI 0.054, 0.672
P=0.0027
Niraparib 8 6 3 0
Vehicle 8 2 1 0
G
Survival probability of HRD- population (PFS)
HR 0.141
95%CI 0.043, 0.463
P=0.00011
Niraparib 12 9 6 2 2
Vehicle 12 1 0 0 0
H
Survival probability of Total population (OS)
HR 0.259
95%CI 0.134, 0.503
P=0.0099
Niraparib 26 16 7 3 2
Vehicle 30 17 3 0 0
I
Survival probability of HRD+ population (OS)
P=0.52
Niraparib 8 6 3 2
Vehicle 6 3 0 0
J
Survival probability of HRD- population (OS)
HR 0.213
95%CI 0.066, 0.691
P=0.0036
Niraparib 12 8 3 2 0
Vehicle 11 3 0 0 0
