# Supplementary material for: Using Patient-Derived Xenograft (PDX) Models as a ‘Black Box’ to Identify More Applicable Patients for ADP-Ribose Polymerase Inhibitor (PARPi) Treatment in Ovarian Cancer: Searching for Novel Molecular and Clinical Biomarkers and Performing a Prospective Preclinical Trial
Source: Cancers (Basel). 2022 Sep 24;14(19):4649. doi: 10.3390/cancers14194649 (PMC9563731; doi:10.3390/cancers14194649)
Supplement: Supplementary file 1 [file cancers-14-04649-s001.zip › Supplementary Table.pdf]

Table S1. Response Category

Response Category	Best Response (%)
CR	<-95%
PR	[-95%, -50%)
PR>SD	[-50%, 0%)
SD	[0%, 35%)
SD>PD	[35%, 100%)
PD	Others

CR,complete remission; PR, partial remission; SD, stable disease; PD, progression disease.

Table S2. Drug efficacy of PDX

No.	Niraparib	Olaparib	TC
1	No	-	Yes
2	No	No	No
3	No	-	Yes
4	Yes	-	No

5	No	Yes	Yes
6	No	-	Yes
7	Yes	Yes	-
8	Yes	-	Yes
9	No	-	Yes
10	Yes	-	Yes
11	No	No	Yes
12	Yes	Yes	-
13	No	-	No
14	Yes	-	-
15	Yes	-	Yes
16	Yes	-	Yes
17	Yes	No	Yes
18	No	No	Yes
19	No	No	-
20	Yes	Yes	Yes
21	Yes	Yes	Yes
22	Yes	Yes	Yes
23	No	No	Yes
24	Yes	Yes	-
25	Yes	Yes	-
26	Yes	Yes	-

27	Yes	Yes	-
28	Yes	Yes	Yes
29	Yes	Yes	Yes
30	Yes	Yes	-
31	No	Yes	-
32	Yes	Yes	No
33	Yes	No	-
34	-	Yes	-
35	Yes	Yes	-
36	No	Yes	-
37	Yes	Yes	Yes
38	No	-	-
39	No	-	-
40	No	No	-

No, invaild (mSD>RD+mPD); Yes, valid (mSD+mPR>SD+mPR+mCR); TC, Paclitaxel and carboplatin.

Table S3. Survival data of patients using PARPi as first- of second-line maintenance therapy.

NO	First-line maintenance therapy			PDX's response	Second-line maintenance therapy			PDX's response
	PARPi	Recur-rent	PFS (Month)		PARPi	Recur-rent	PFS (Month)	
2	niraparib	Yes	10	Non-Response	niraparib	Yes	6	Non-Response

5	olaparib	No	42	Response	-	-	-	-
7	olaparib	Yes	38	Response	-	-	-	-
8	olaparib	No	16	Response	-	-	-	-
15	niraparib	Yes	21	Response	olaparib	No	24	Response
16	olaparib	Yes	21	Response	olaparib	Yes	18	Response
17	niraparib	No	37	Response	-	-	-	-
18	niraparib	Yes	13	Non-Response	olaparib	Yes	16	Non-Response
20	niraparib	Yes	28	Response	olaparib	No	10	Response
21	niraparib	No	27	Response	-	-	-	-
22	olaparib	Yes	23	Response	niraparib	Yes	12	Response
24	olaparib	No	27	Response	-	-	-	-
29	-	-	-	-	olaparib	Yes	17	
30	niraparib	Yes	16	Response	-	-	-	-
31	-	-	-	-	niraparib	No	8	Non-Response
37	-	-	-	-	olaparib	No	7	Response

Table S4. The comparison of gene amplification between response and non-response groups.

**Gene**	***P-value***	**Odds_ratio**	**Percentage_Response**	**Percentage_No_Response**	***FDR***
PAX8	*0.116615*	inf	0.333333	0	*1*
NKX2-1	*0.116615*	inf	0.333333	0	*1*
SOX2	*0.167468*	5	0.75	0.375	*1*

CDKN2A	*0.324561*	5	0.416667	0.125	*1*
BRCA1	*0.347265*	0.333333	0.166667	0.375	*1*
MYC	*0.347265*	3	0.833333	0.625	*1*
MDM2	*0.356347*	0.333333	0.25	0.5	*1*
CCND1	*0.356347*	0.333333	0.25	0.5	*1*
CDKN1B	*0.372851*	0.333333	0.5	0.75	*1*
NF1	*0.4*	0	0	0.125	*1*
STK11	*0.494737*	inf	0.166667	0	*1*
ERBB2	*0.536842*	0.272727	0.083333	0.25	*1*
ATM	*0.642415*	0.555556	0.25	0.375	*1*
CDK4	*0.647916*	0.5	0.333333	0.5	*1*
BCL2L1	*0.647916*	2	0.666667	0.5	*1*
KRAS	*0.647916*	2	0.666667	0.5	*1*
E2F3	*0.669921*	0.6	0.5	0.625	*1*
IKZF2	*0.669921*	1.666667	0.5	0.375	*1*
MLL3	*1*	1	0.5	0.5	*1*

Table S5. The comparison of gene deletion between response and non-response groups.

**Gene**	***P-value***	**Odds_ratio**	**Percentage_ Response**	**Percentage_No_ Response**	***FDR***
AKT1	*0.014654*	inf	0.583333	0	*0.5129*
KRAS	*0.049123*	0	0	0.375	*0.566802*
NF1	*0.062332*	8.333333	0.833333	0.375	*0.566802*

SMAD4	*0.064777*	9	0.75	0.25	*0.566802*
PDGFRA	*0.161094*	0.2	0.166667	0.5	*0.837342*
ERBB2	*0.161094*	5	0.833333	0.5	*0.837342*
CDKN2A	*0.167468*	0.2	0.25	0.625	*0.837342*
MDM4	*0.347265*	0.333333	0.166667	0.375	*1*
CCND1	*0.494737*	inf	0.166667	0	*1*
IGF1R	*0.536842*	0.272727	0.083333	0.25	*1*
MYCN	*0.536842*	0.272727	0.083333	0.25	*1*
CCNE1	*0.536842*	0.272727	0.083333	0.25	*1*
E2F3	*0.536842*	0.272727	0.083333	0.25	*1*
NEDD9	*0.536842*	0.272727	0.083333	0.25	*1*
CDK4	*0.602683*	3.5	0.333333	0.125	*1*
STK11	*0.618576*	0.428571	0.75	0.875	*1*
MLL3	*0.618576*	2.333333	0.25	0.125	*1*
CDK6	*0.618576*	2.333333	0.25	0.125	*1*
FAT1	*0.618576*	0.428571	0.75	0.875	*1*

Table S6. The comparison of gene mutation between response and non-response groups.

**Gene**	**Odds_ratio**	***P-value***	**Percentage_Response**	**Percentage_No_Response**	***FDR***
SYMPK	0.04	*0.147368*	0.0001	0.25	*1*
SELP	0.04	*0.147368*	0.0001	0.25	*1*
COL6A5	0.04	*0.147368*	0.0001	0.25	*1*

ABCB5	0.04	*0.147368*	0.0001	0.25	*1*
MTCL1	0.04	*0.147368*	0.0001	0.25	*1*
NF2	0.04	*0.147368*	0.0001	0.25	*1*
MUTYH	0.04	*0.147368*	0.0001	0.25	*1*
LRRK2	25	*0.242105*	0.25	0.0001	*1*
SI	25	*0.242105*	0.25	0.0001	*1*
EYS	25	*0.242105*	0.25	0.0001	*1*
KDM6A	0.04	*0.4*	0.0001	0.125	*1*
GRIN3A	0.04	*0.4*	0.0001	0.125	*1*
MS4A12	0.04	*0.4*	0.0001	0.125	*1*
GSG2	0.04	*0.4*	0.0001	0.125	*1*
HIST1H4D	0.04	*0.4*	0.0001	0.125	*1*
MYCBP2	0.04	*0.4*	0.0001	0.125	*1*
CYP2A13	0.04	*0.4*	0.0001	0.125	*1*
GLTPD2	0.04	*0.4*	0.0001	0.125	*1*
TRPM1	0.04	*0.4*	0.0001	0.125	*1*
