# Supplementary figures and images for: Using Patient-Derived Xenograft (PDX) Models as a ‘Black Box’ to Identify More Applicable Patients for ADP-Ribose Polymerase Inhibitor (PARPi) Treatment in Ovarian Cancer: Searching for Novel Molecular and Clinical Biomarkers and Performing a Prospective Preclinical Trial
Source: Cancers (Basel). 2022 Sep 24;14(19):4649. doi: 10.3390/cancers14194649 (PMC9563731; doi:10.3390/cancers14194649)

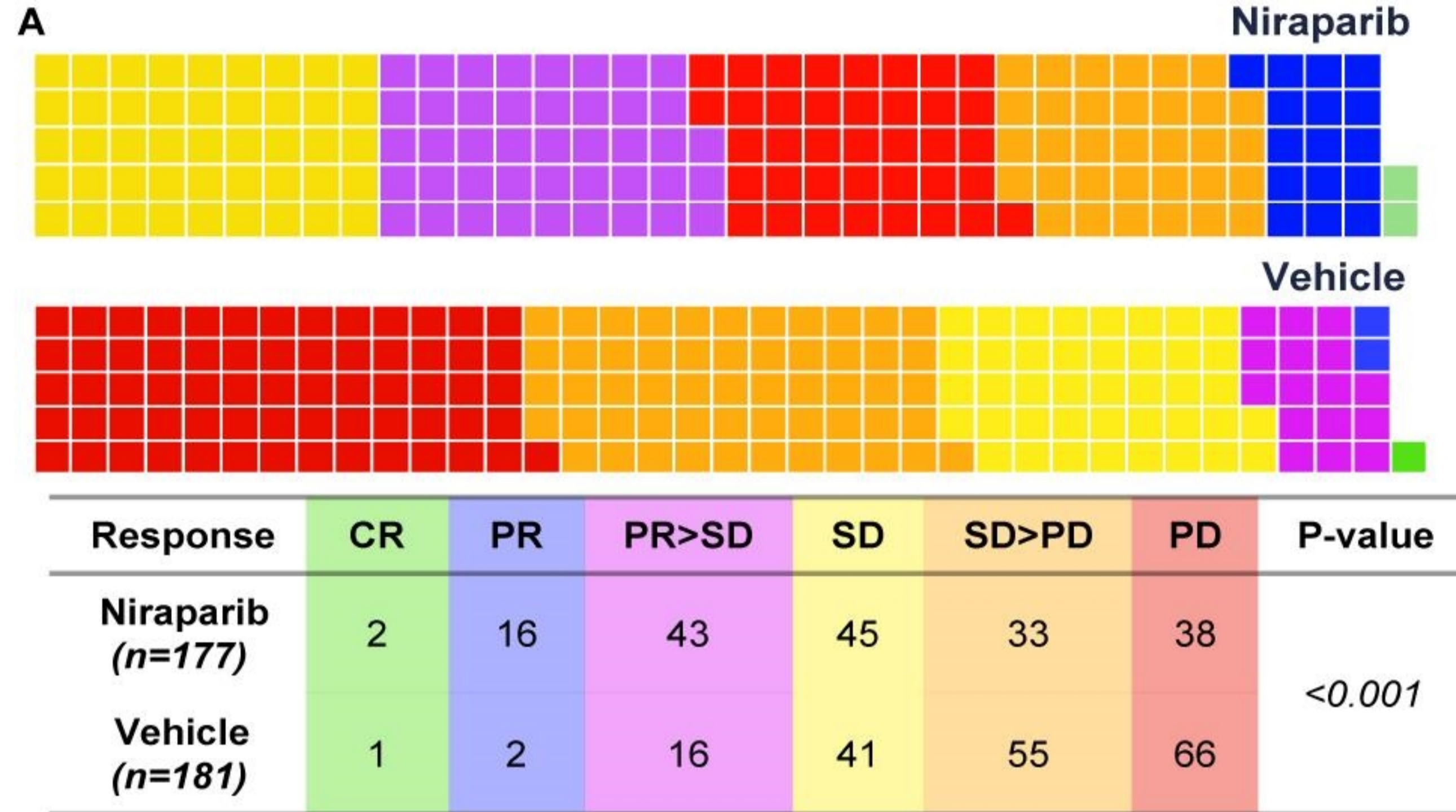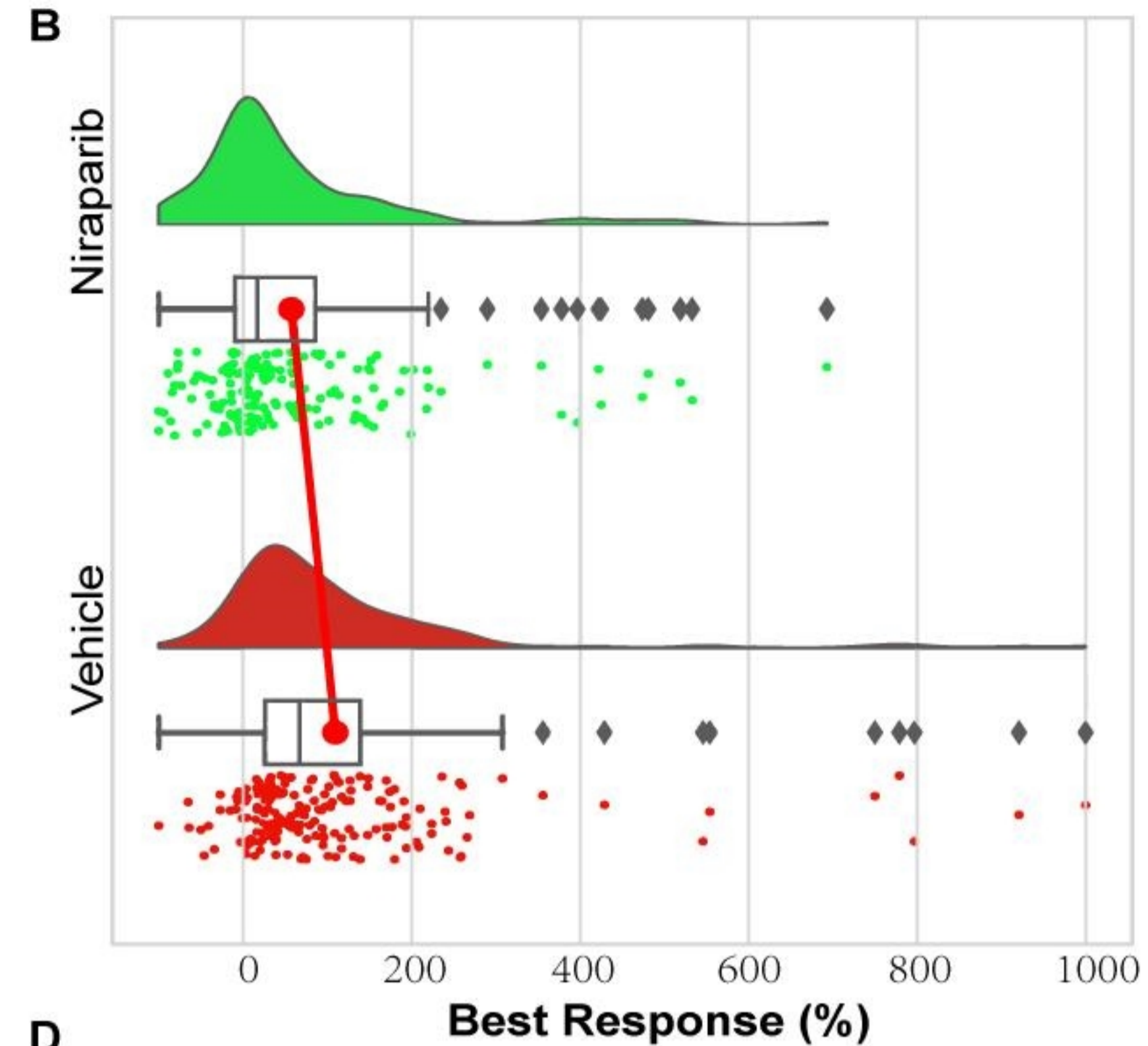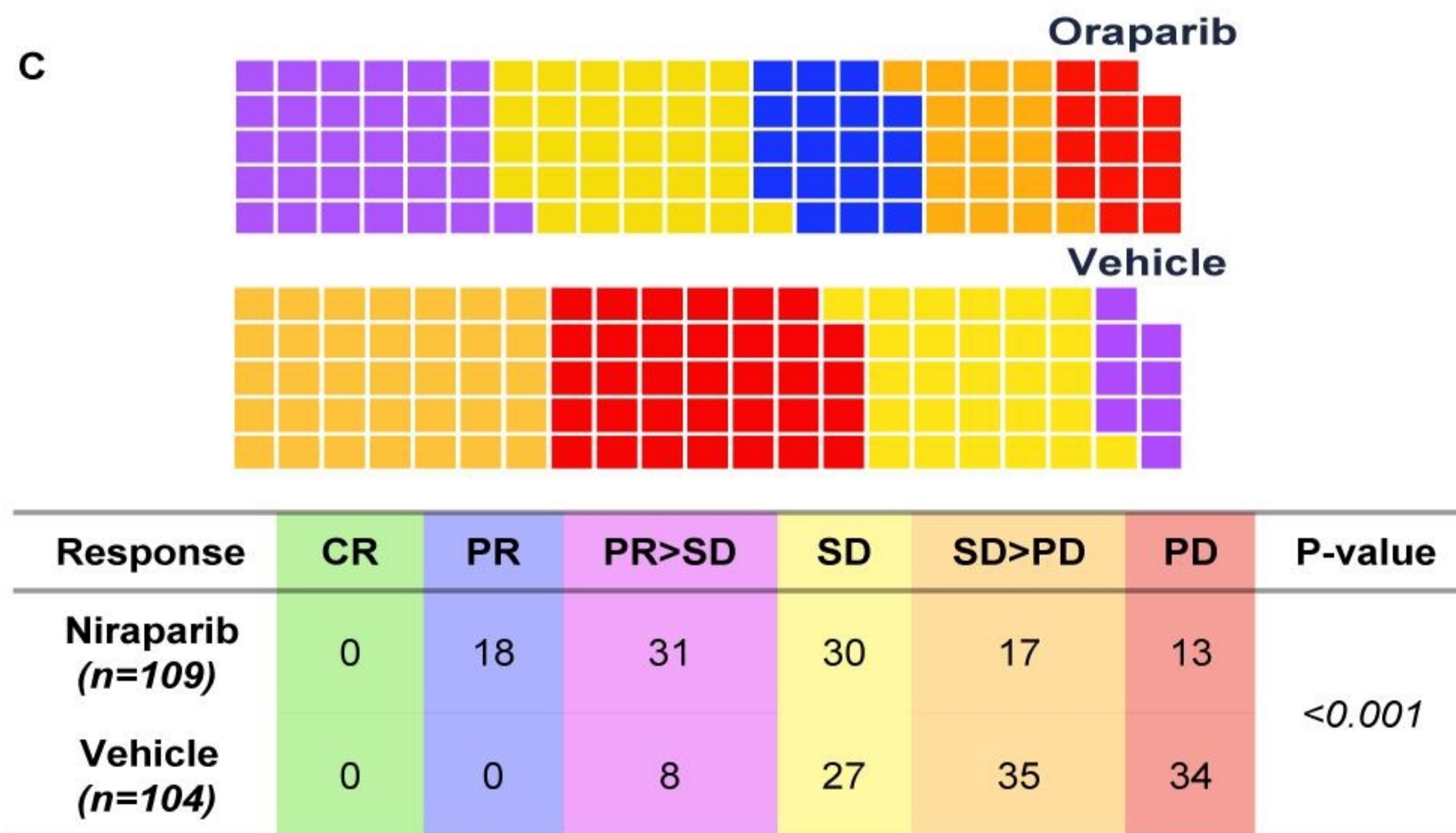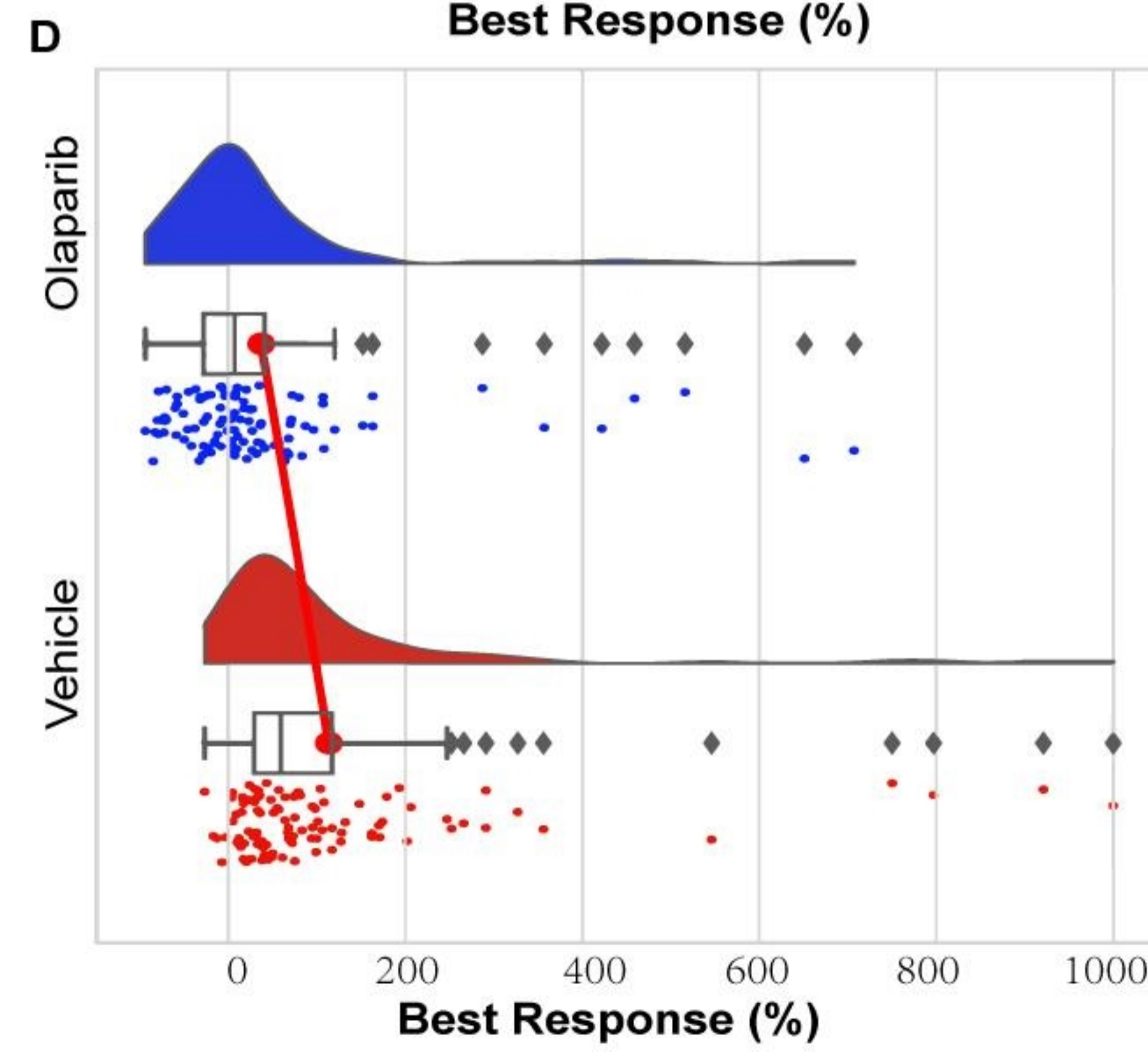

Supplement: Supplementary file 1 [file cancers-14-04649-s001.zip › Supplementary Figure S1.pdf]

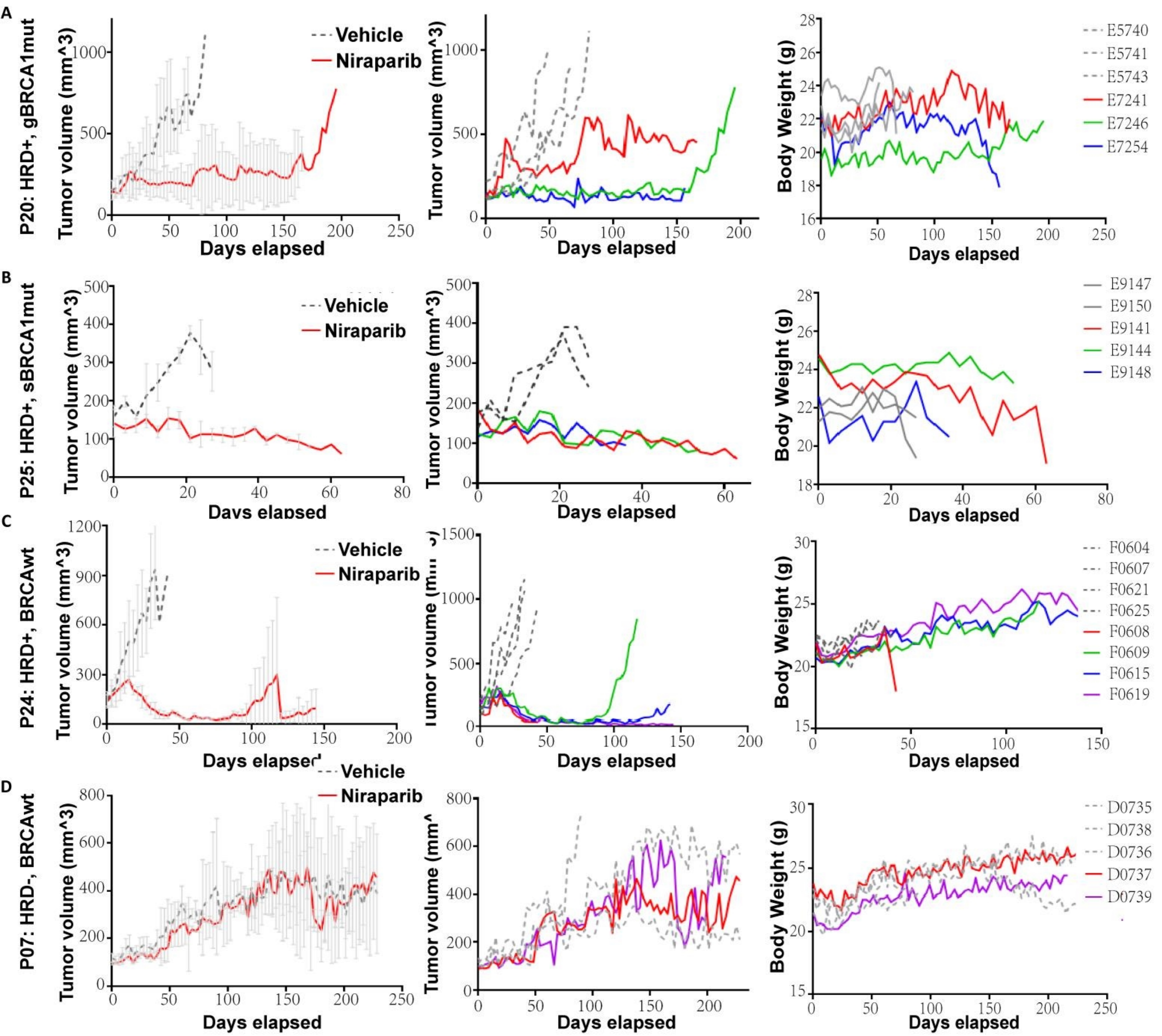

Supplement: Supplementary file 1 [file cancers-14-04649-s001.zip › Supplementary Figure S2.pdf]

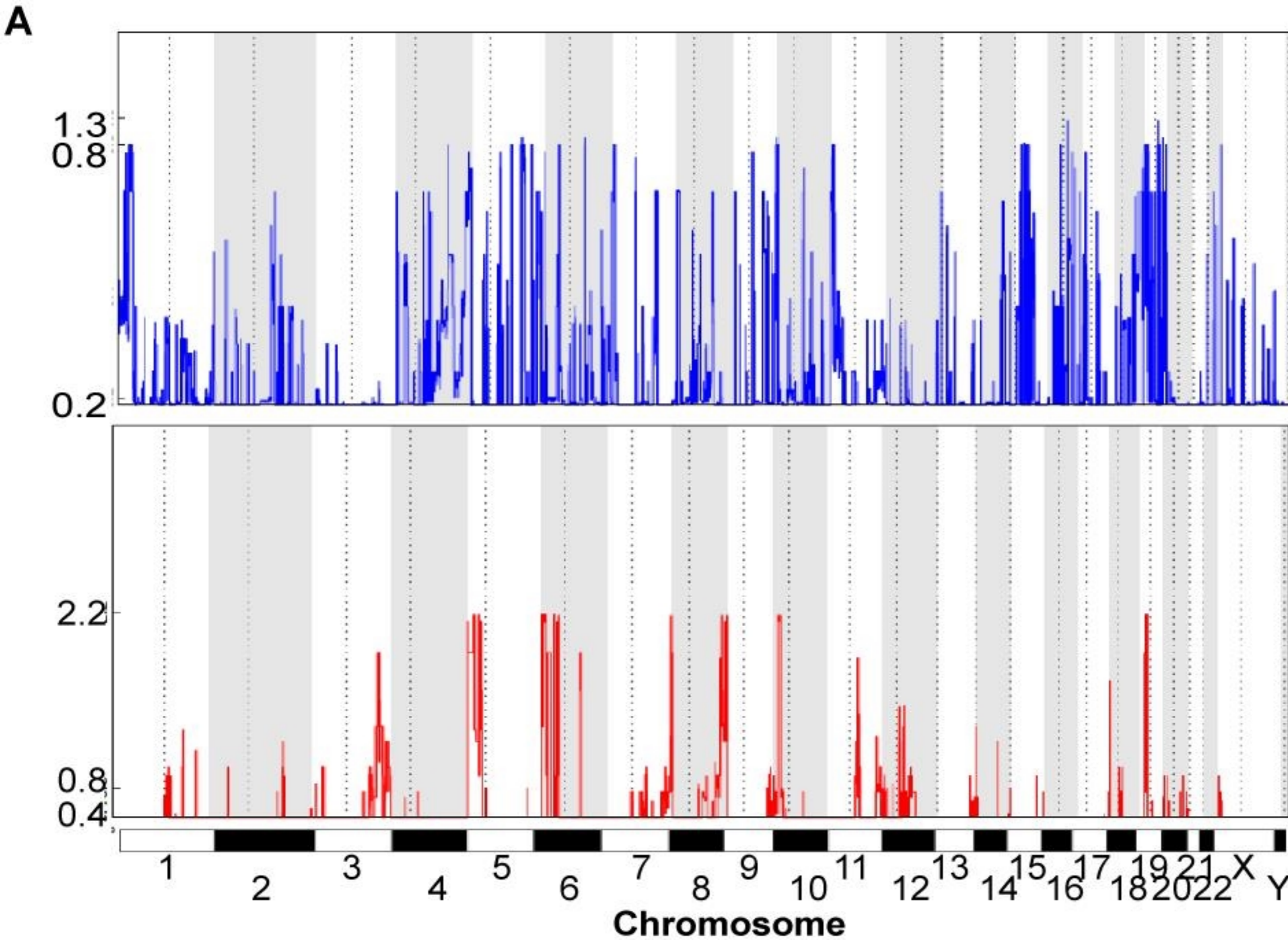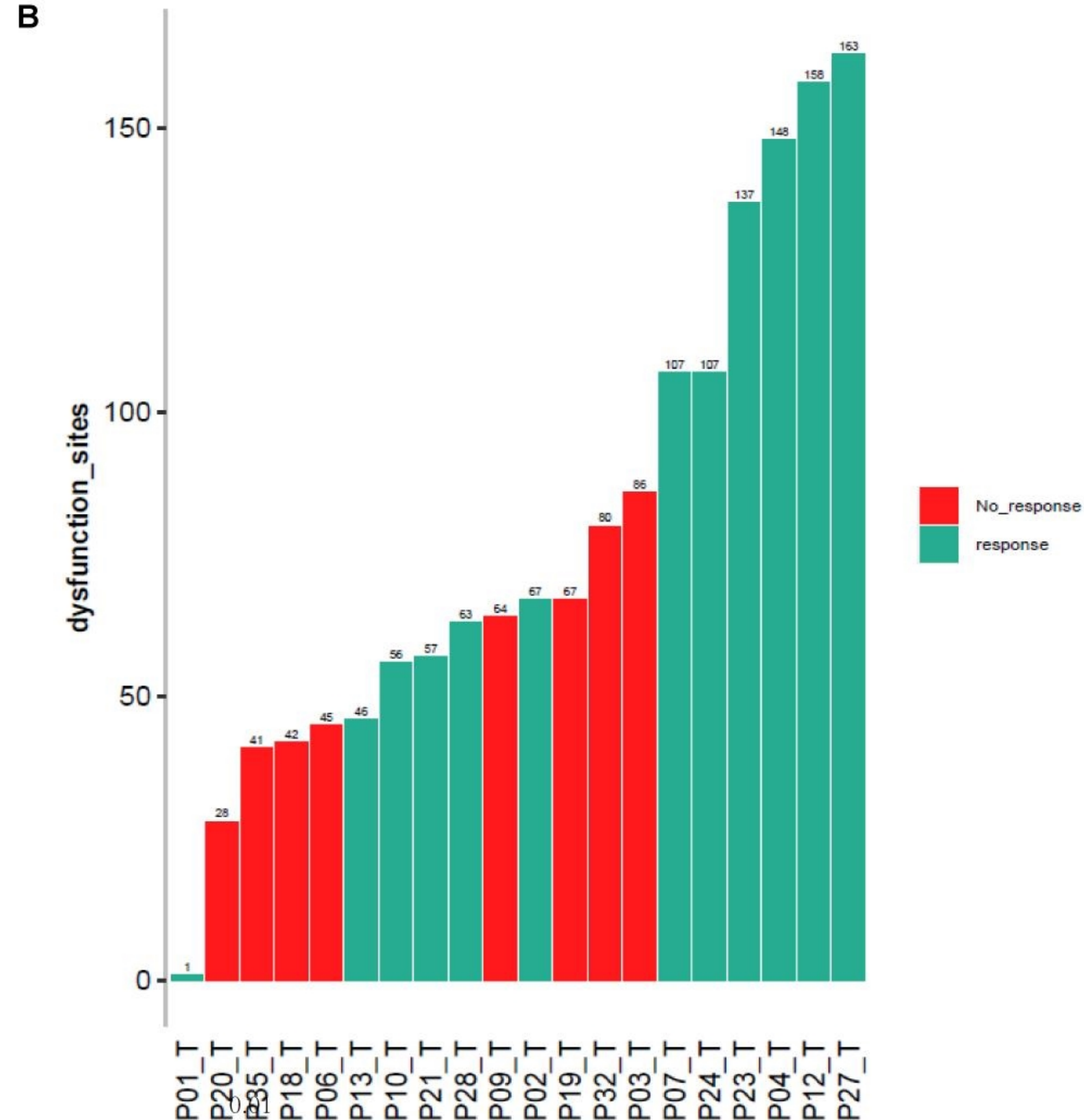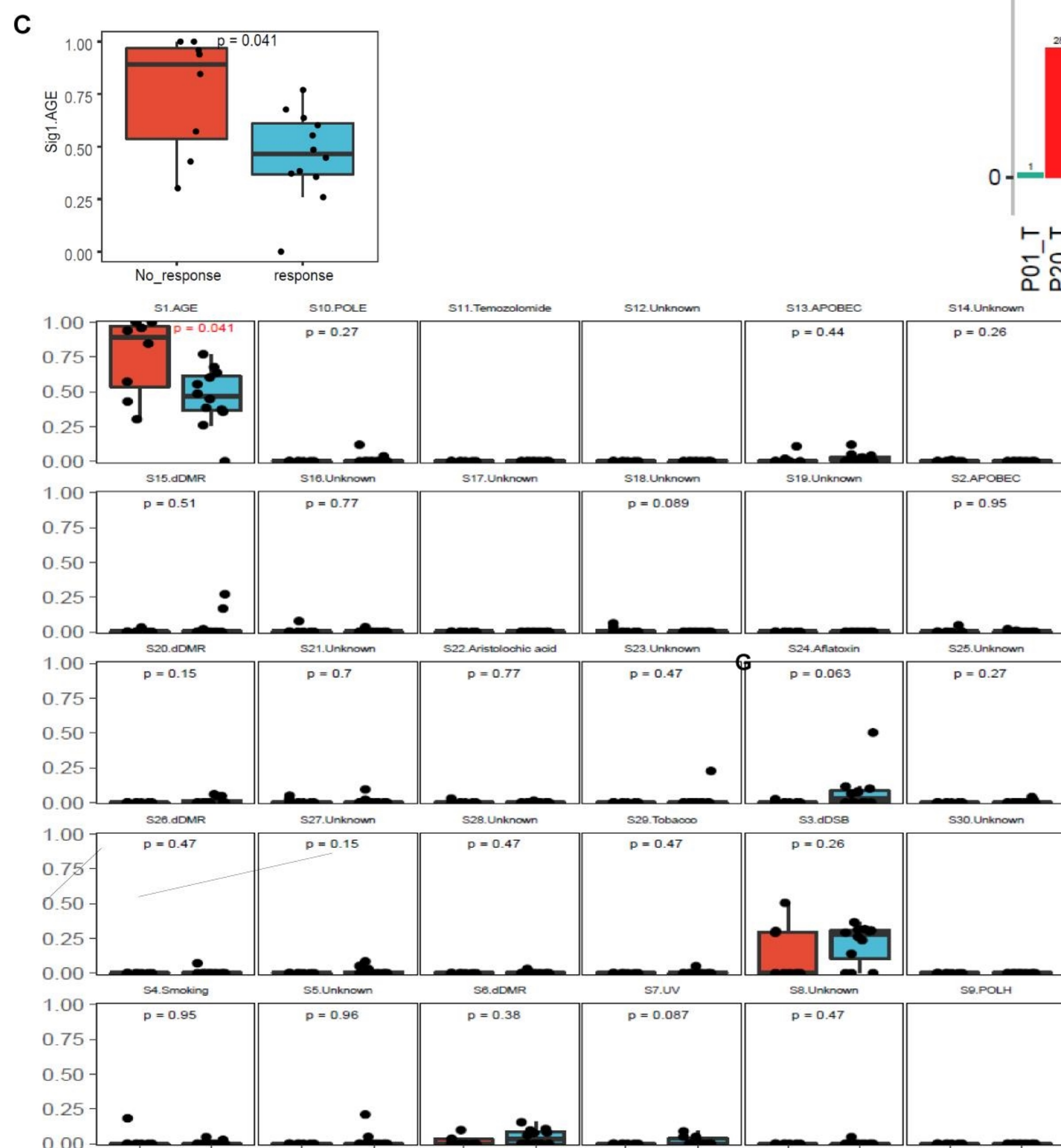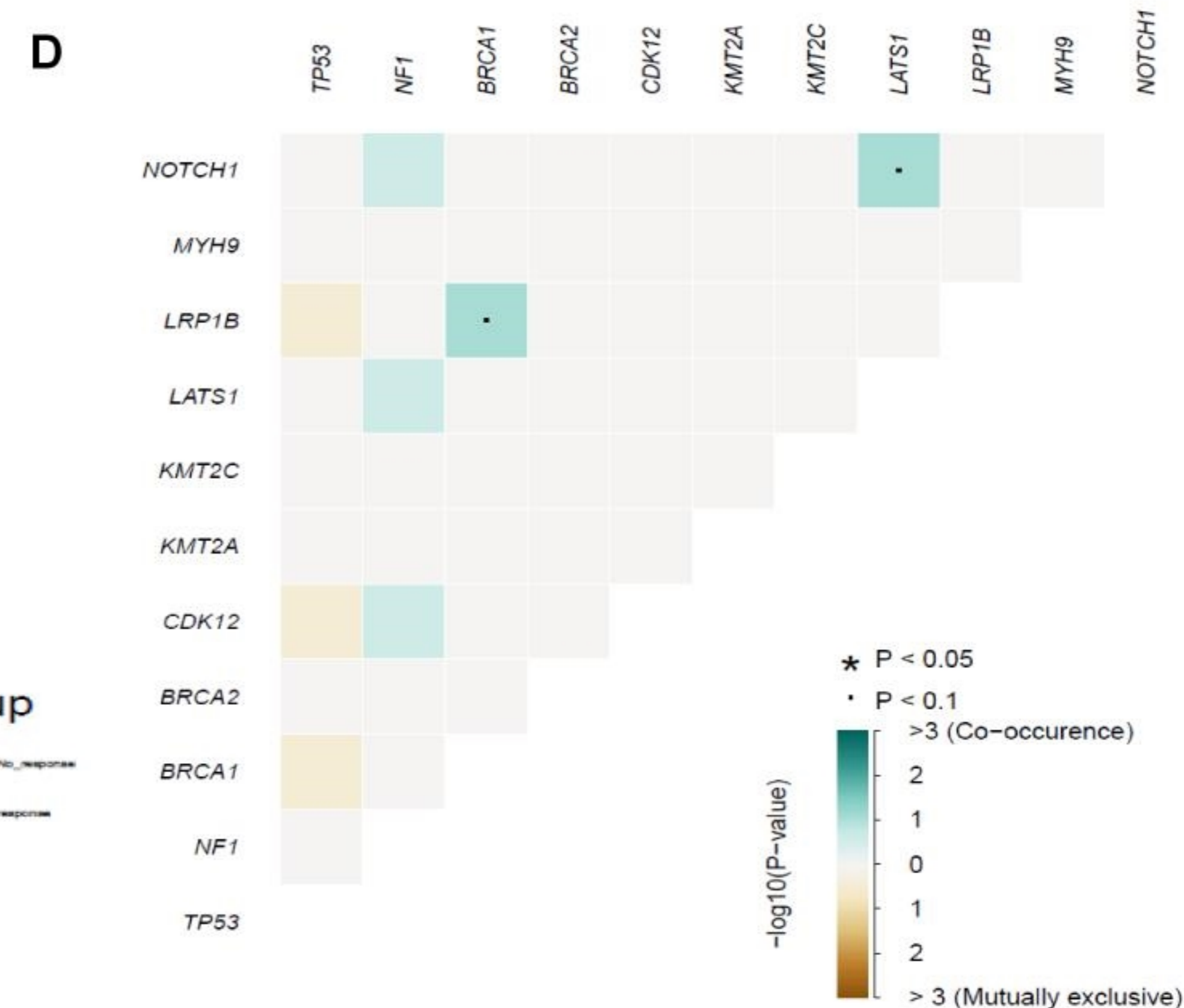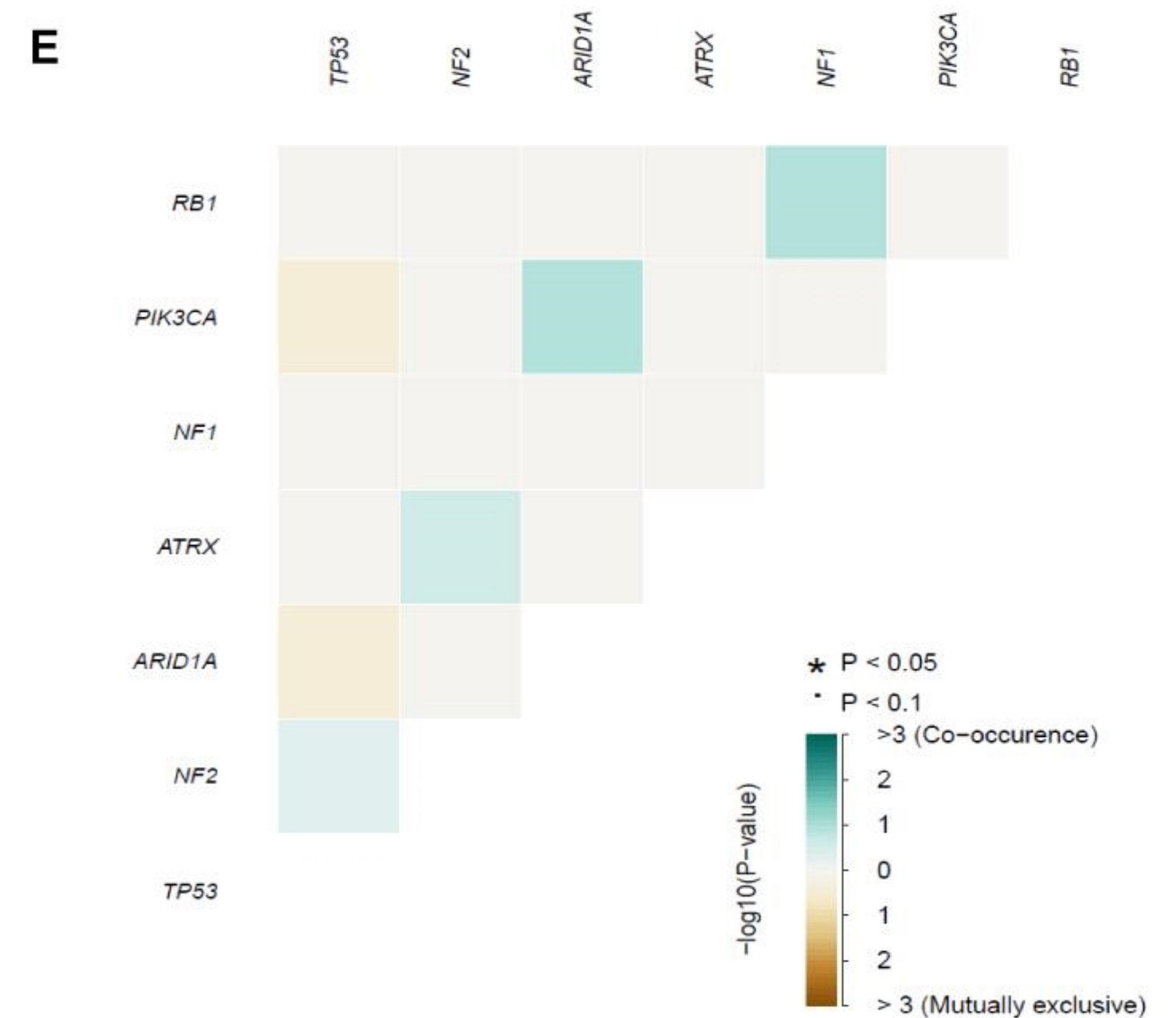

Supplement: Supplementary file 1 [file cancers-14-04649-s001.zip › Supplementary Figure S3.pdf]

**A**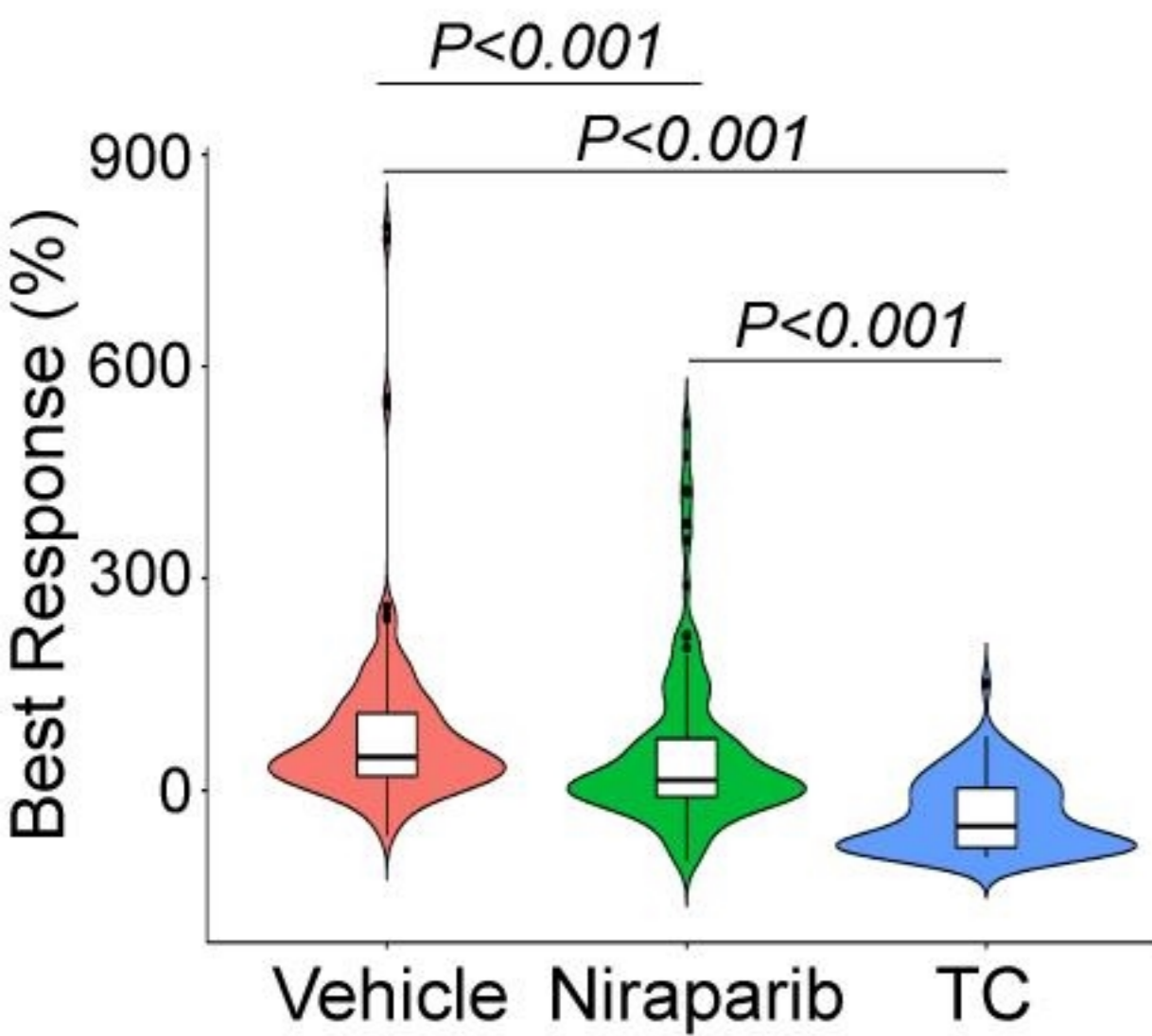**B**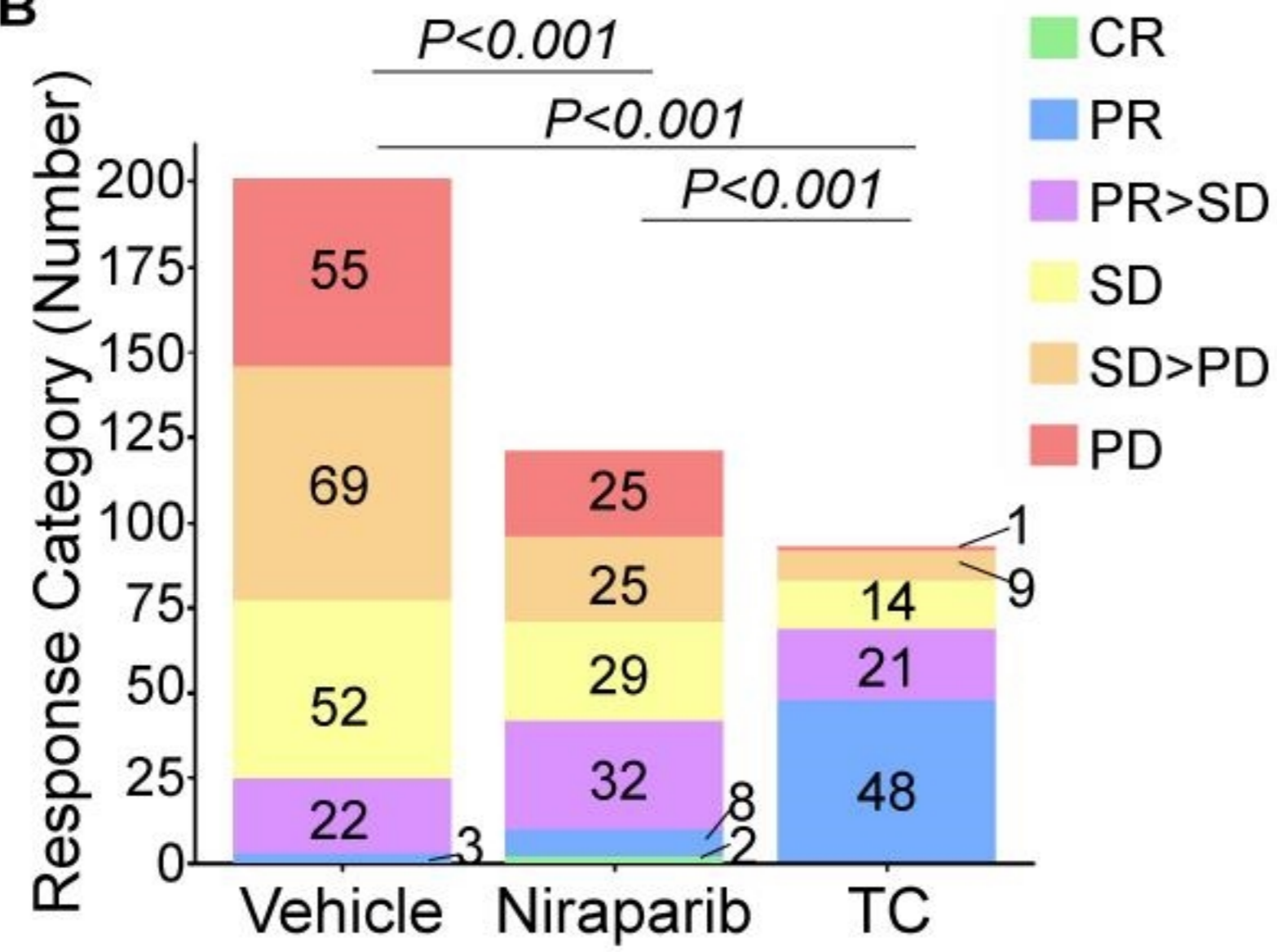**C**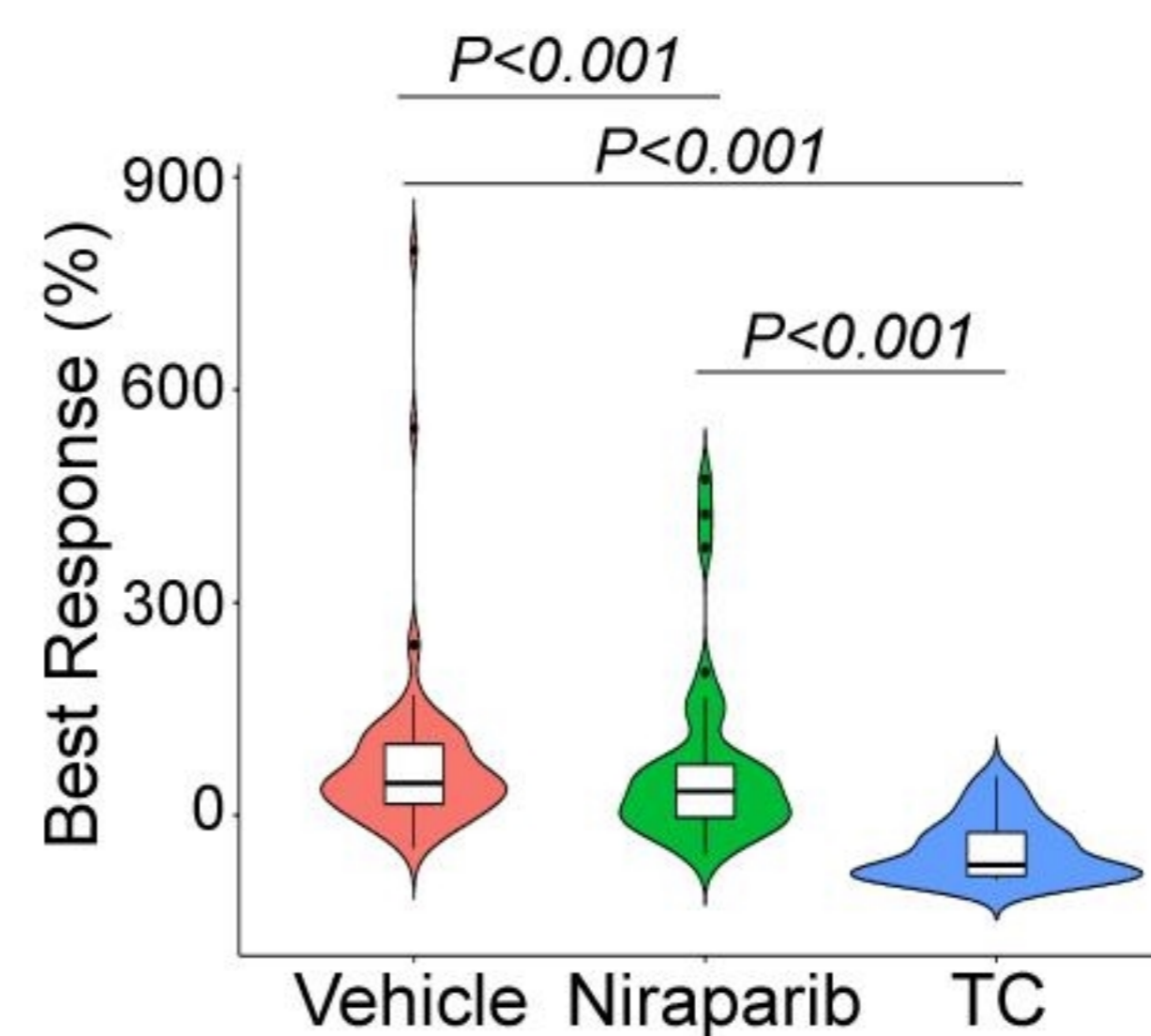**D**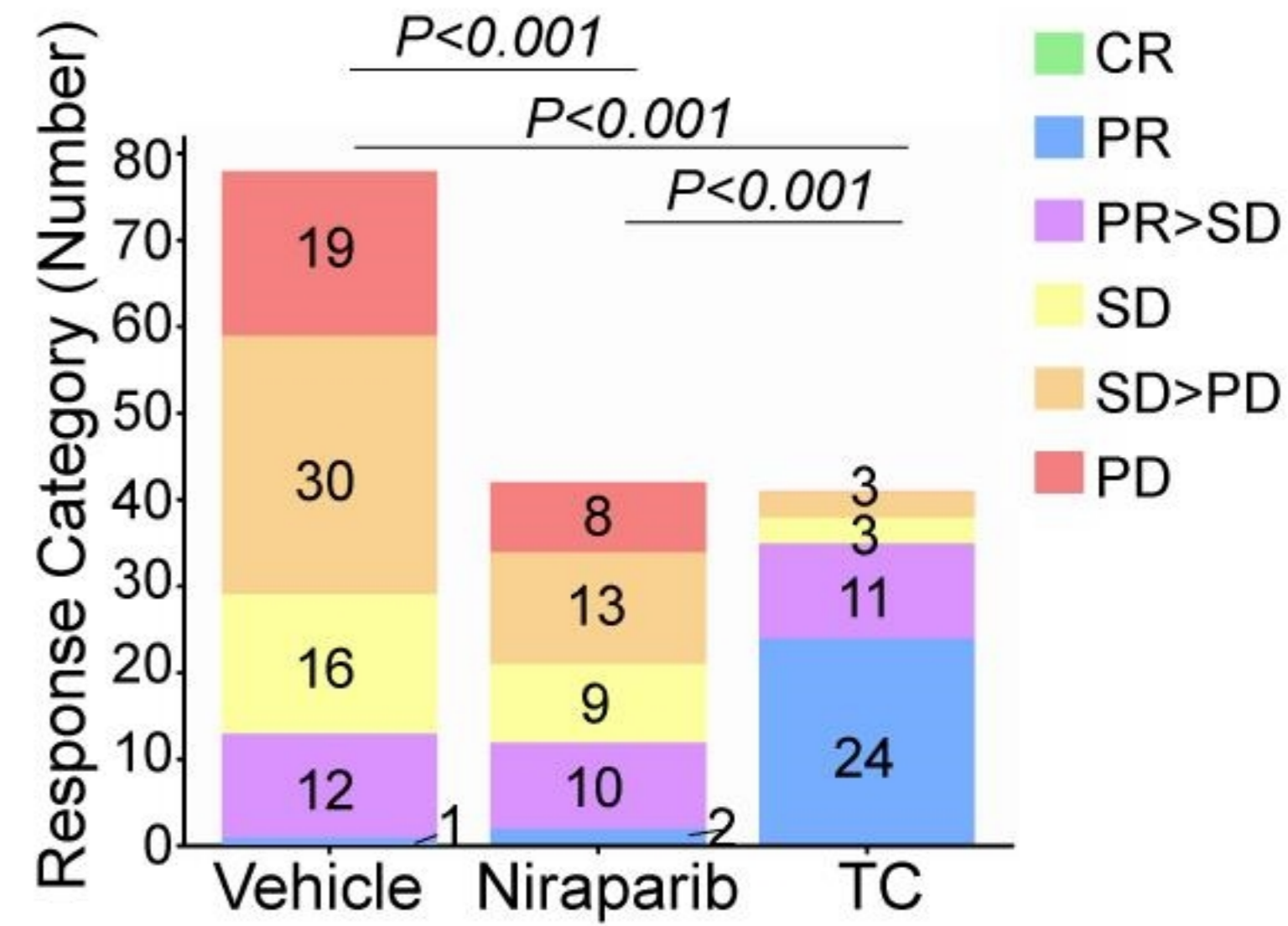**E**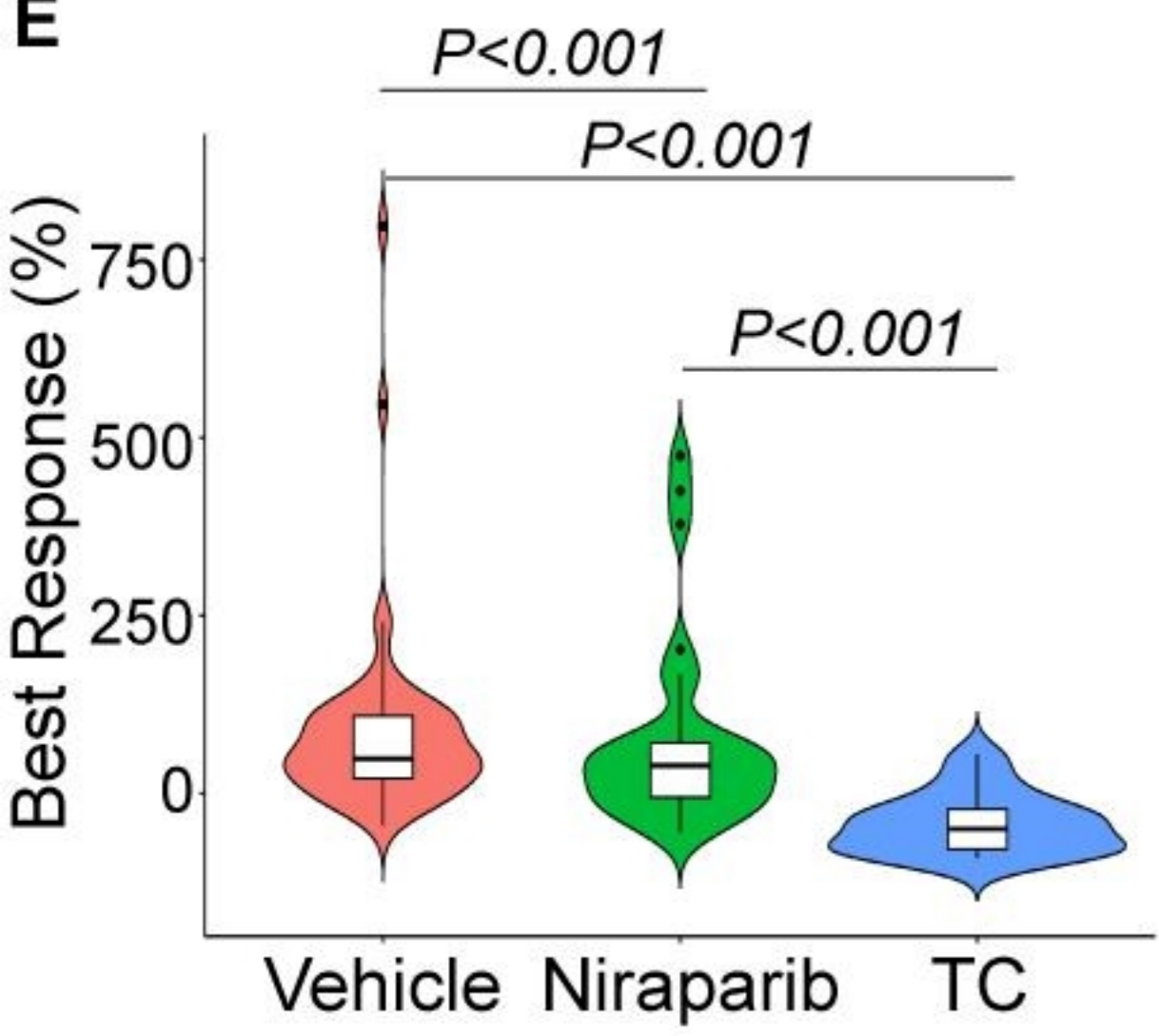**F**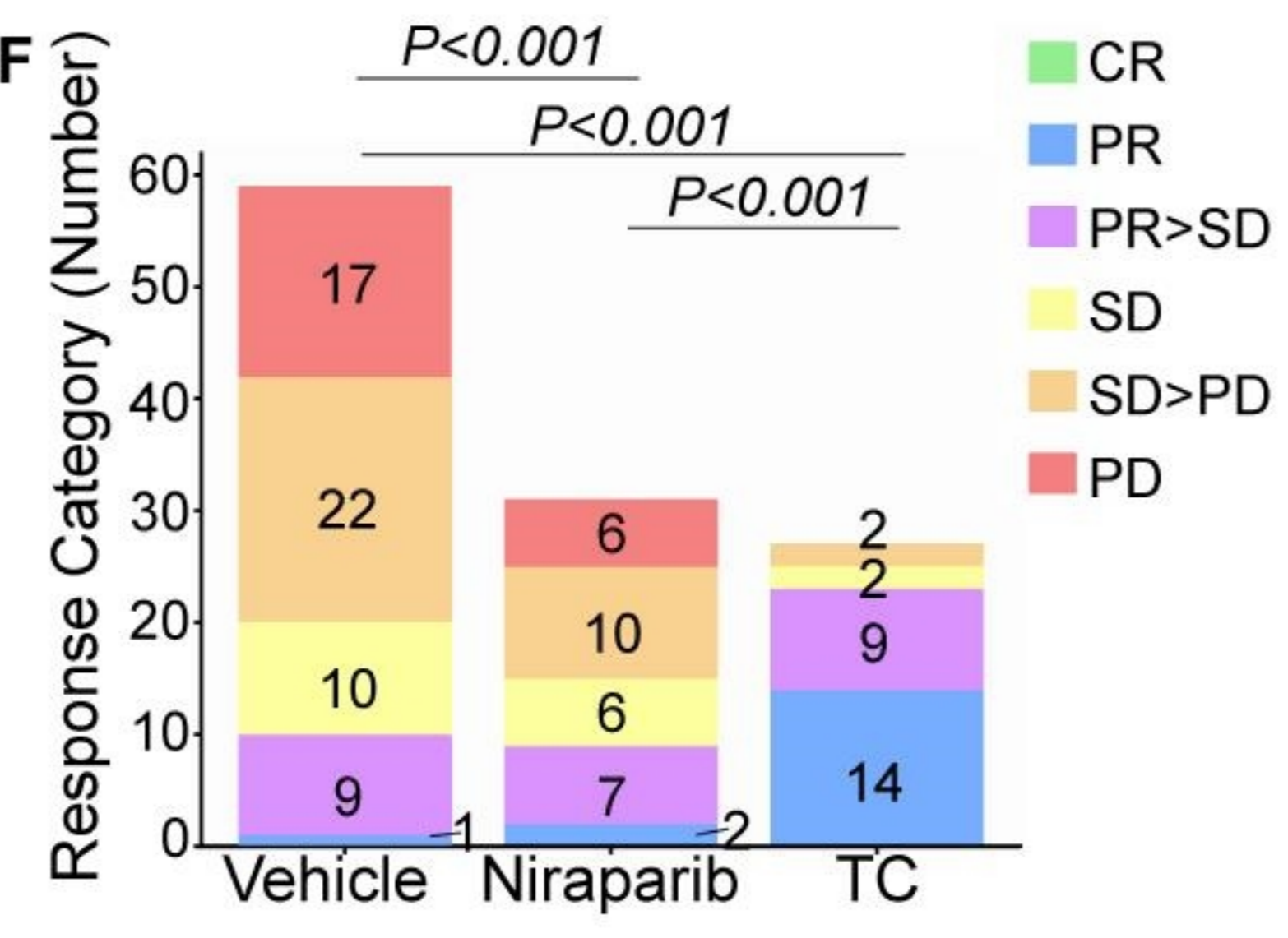**G**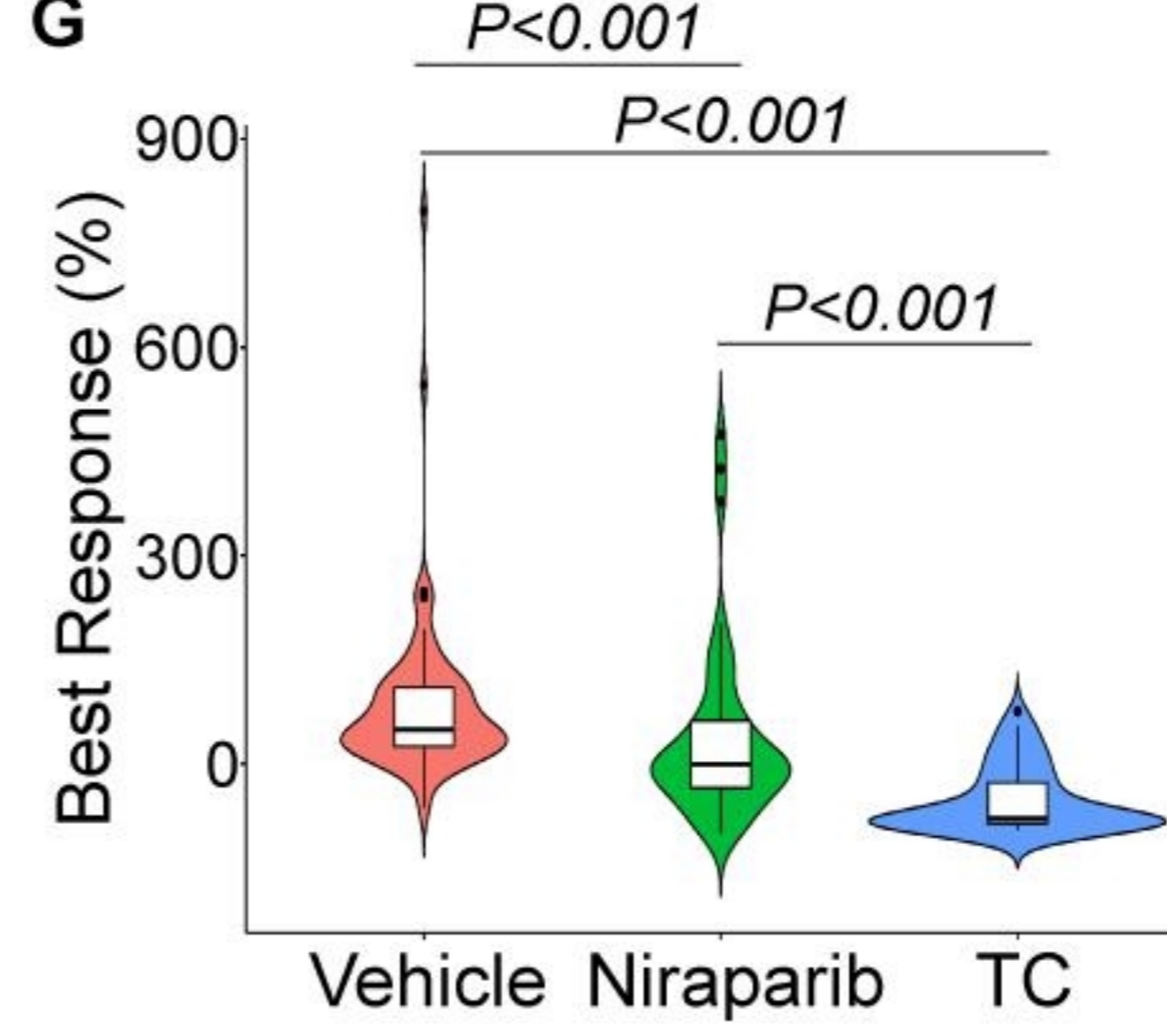**H**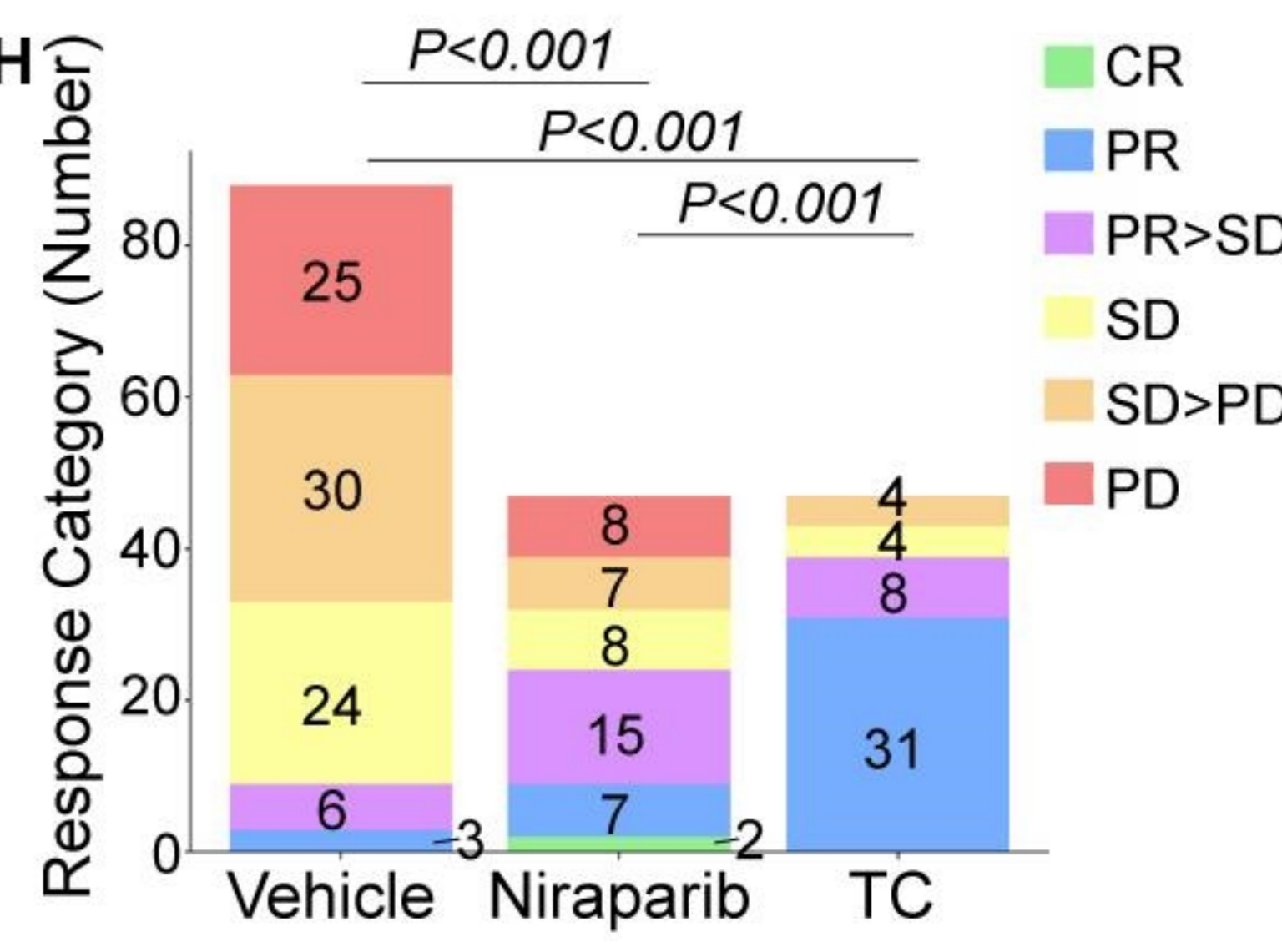

Supplement: Supplementary file 1 [file cancers-14-04649-s001.zip › Supplementary Figure S5.pdf]
